# Persistent deficits in knee joint kinematics and kinetics during gait following tibial plateau fractures – a longitudinal study

**DOI:** 10.1186/s12891-024-07910-3

**Published:** 2024-10-14

**Authors:** Anna Fändriks, Roland Zügner, Jón Karlsson, Michael Möller, Roy Tranberg

**Affiliations:** Department of Orthopaedics, Institute of Clinical Sciences, Sahlgrenska Academy, University of Gothenburg, SE-431 30 Gothenburg, Sweden

**Keywords:** Tibial plateau fracture, Knee joint, Gait analysis, Kinematics, Kinetics

## Abstract

The recovery process after tibial plateau fractures varies, with some patients experiencing persistent gait asymmetries for a long period of time. The aim of this study is to analyse knee joint kinematics and kinetics post-fracture using a linear mixed-effects model, assessing 26 participants over 24 months (aged 45, range 26–63), and an age-matched control group (aged 47, range 26–62). Participants underwent three-dimensional gait analysis at 6-, 12- and 24-months post-injury. Controls participated in the gait analysis on one occasion. Six gait variables related to knee joint kinematics and kinetics were analysed with a linear mixed-effects model. The model was constructed to determine if there was a differential improvement over time between the injured and the non-injured legs across the six variables, referred to as an interaction effect. If no interaction effect was observed, the model assessed whether there was a side difference between the legs and if there was any improvement over time in both legs. Additionally, non-parametric tests were performed to assess differences between the non-injured leg and the control group across the six variables 24 months after injury. The findings revealed an interaction effect in terms of cumulative absorbed power (*p* = 0.02, side difference *p* = 0.06). Other variables showed no interaction effects. Although a side difference between legs was observed for all variables (*p* < 0.001), only the variables regarding generated power exhibited improvements over time (*p* = 0.02 respectively). Minimal knee flexion, range of motion, and maximal extending knee joint moment showed no improvements over time. At the 24-month follow-up, the maximal extending knee joint moment was the only variable that differed between the non-injured leg and controls, with increased moment observed for the non-injured leg compared with the controls (*p* = 0.03). Taken together, two years post-fracture, patients demonstrated pronounced side differences between the injured and non-injured legs with worse ability to extend the knee joint and to generate power in the injured leg. While the kinetic variables improved over time, there were no improvements observed in kinematic variables. Moreover, the non-injured leg performed similarly to healthy controls in terms of minimal knee flexion, range of motion, and generated and absorbed power.

## Introduction

The recovery process following a tibial plateau fracture is known to vary among patients [1–5]. Gait asymmetries, especially in terms of spatiotemporal parameters have been observed to persist for a long period of time; even up to three years after the injury in many patients [6–9]. However, studies with follow-up periods exceeding three years have not shown any significant gait asymmetries [4, 10, 11]. Although improvements in knee joint range of motion (ROM) during walking have been reported between six and twelve months [12], differences between the injured and non-injured sides have been noted to persist even two years after injury [13]. Improvements towards normal joint loading have mainly been reported to occur between three and six months after the injury [14].

The commonly used approaches to assess gait function in previous studies have involved a pressure-sensitive mat, measuring spatiotemporal parameters such as cadence, speed, and step length [4, 7, 10, 11], a mobile device to measure joint angles [12], a walking platform with integrated force plates [6] and camera-based three-dimensional motion analysis for detailed objective analysis of gait function [13–15].

To our knowledge only two previous studies have provided longitudinal data in terms of kinematics and kinetics after a tibial plateau fracture [13, 14]. Since walking plays a crucial role in maintaining independence in daily life, it is crucial to assist patients in regaining as optimal walking function after injury as possible.

This study aimed to investigate knee joint kinematics and kinetics during gait over time in both the injured and the non-injured legs following a unilateral tibial plateau fracture. To achieve this objective, a selection of six gait variables associated with the knee joint, derived from three-dimensional motion analysis, were utilized. The two kinematic variables, included for analysis, were minimal knee flexion during single stance together with range of motion (ROM) during gait cycle for flexion–extension in the knee joint. The four kinetic variables, included for analysis, were maximal knee joint extending moment during single stance, maximal generated knee joint power during stance and the cumulative sum of generated and absorbed knee joint power respectively during gait cycle. Subsequently, a linear mixed-effects model was fitted to characterize the expected values and variability for these six variables. The linear mixed-effects model considered both *the effect of time after injury* and *the effect of knee injury status* – indicating whether the leg was injured or not – as well as the interaction effect between these two factors. By separately examining both main effects and their interaction, the aim was to obtain a comprehensive understanding of how time and knee injury status influence knee joint kinematics and kinetics. The second aim was to examine whether the non-injured leg was affected by the injury at the 24-months follow-up through a comparison with a gender- and age-matched healthy control group.

## Methods

### Participants

This longitudinal cohort study included patients who had experienced a tibial plateau fracture and were treated at a university hospital. Inclusion criteria were age between 18 and 65 years old at the time of injury and a unilateral proximal tibia fracture classified as type B or C according to the AO foundation/Orthopaedic Trauma Association (AO-OTA) 2007 fracture classification [16]. Participants with multiple fractures, previous injury or surgery to the lower extremities or any other pre-existing condition affecting their gait function (e.g. neurological disturbances) were excluded. Cognitive disorder was also considered as exclusion criteria. Subjects were verbally invited to participate 2–5 months after the injury, followed by oral and written information about the study.

The study period spanned 17 months, between 2018 and 2020. During this period 43 participants were identified. Six declined participation and eleven could not be contacted. Finally, 26 participants were included in the study (Table 1). Follow-up assessments were conducted at six months, 12 months, and 24 months following the injury. At the six months follow-up, participants had a median age of 45 years (range 26–63), body mass index (BMI) of 25.7 (inter quartile range (IQR) 5.45) and a walking speed of 1.27 m/s (IQR 0.2). BMI and walking speed changed slightly until the two-year follow-up (Table 1). One participant declined participation at the 12 months follow-up and 6 participants declined participation at 24 the months follow-up (Table 1). Data from one participant were deemed unusable for analysis during the 24 months follow-up due to technical issues.

**Table 1:** Characteristics of included patients with tibial plateau fractures and matched controls

	**6 months** *n* = 26	**12 months** *n* = 25	**24 months** *n* = 18	**Controls** *n* = 36
Female, n (%)	13 (50%)	12 (48%)	9 (50%)	18 (50%)
Injured side left, n (%)	21 (84%)	21 (84%)	15 (79%)	-
Surgical treatment, n (%)	20 (77%)	20 (80%)	14 (78%)	-
Age, years (range)	45 (26–63)	45 (27–63)	47 (28–64)	47 (26–62)
Height, m (IQR)	1.74 (0.13)	1.75 (0.2)	1.75 (0.1)	1.75 (0.1)
Bodyweight, kg (IQR)	75.6 (19.4)	74.1 (16.6)	80.5 (18)	75.0 (17.9)
BMI, m^2^/kg (IQR)	25.7 (5.5)	25.1 (4.8)	26.5 (7.8)	24.8 (5.2)
Speed, m/s (IQR)	1.3 (0.2)	1.3 (0.2)	1.3 (0.2)	1.3 (0.2)
Fracture classification according to AO/OTA				
B1, n	6	6	5	
B2, n	6	5	3	
B3, n	5	5	4	
C1, n	3	3	2	
C3, n	5	5	4	
Unknown, n	1	1	0	

Data are shown as number (n) of participants (%) or median (range or IQR). *BMI*  Body Mass Index, *AO/OTA*AO foundation/Orthopaedic Trauma Association

Participants followed individualized postoperative rehabilitation protocols according to hospital routine, which included a period of non-weightbearing in the initial phase after injury, follow-up appointments with orthopaedic surgeons and visits with physiotherapists within or outside the hospital’s regimen.

The reference group consisted of 36 gender- and age-matched (± 5 years) controls matching the 18 participants during the 24-months follow-up (ratio 2:1) (Table 1). Data from the right leg of the reference group was used.

### Data collection

**Fig. 1:**
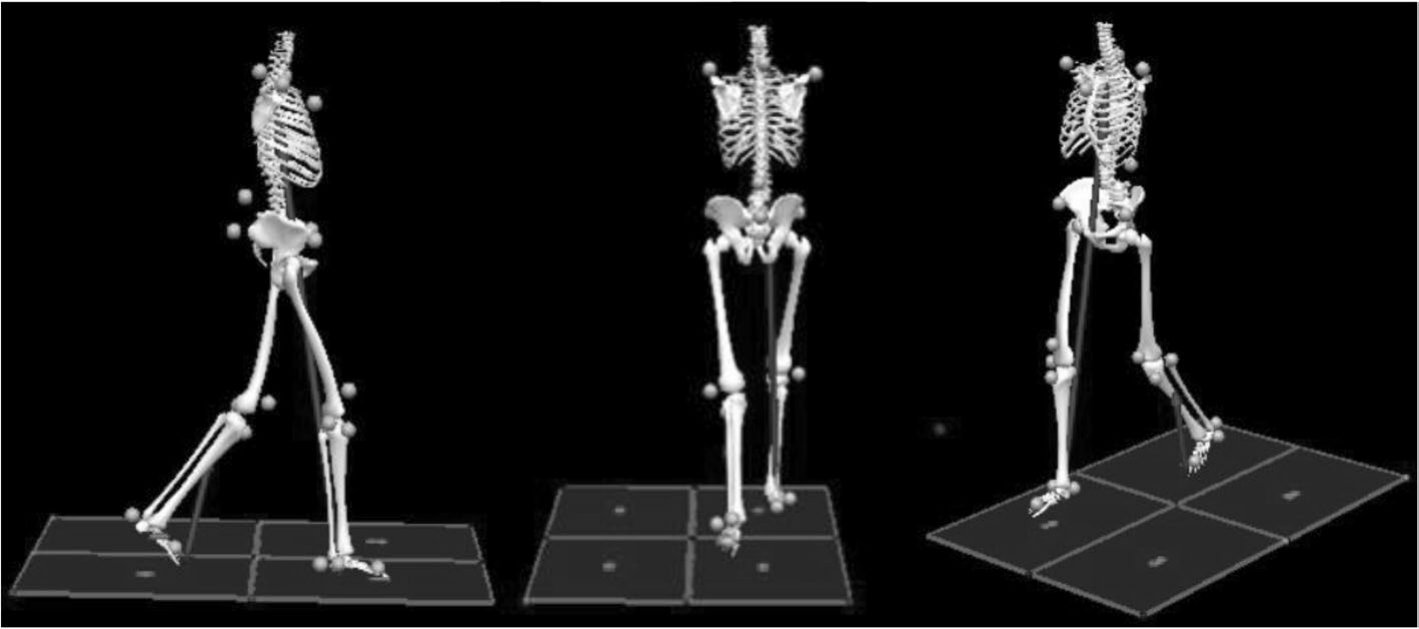
Illustration from the three-dimensional gait analysis from one participant. A marker set with twenty reflective markers was used, including the trunk, pelvis, and leg segments. The grey arrows indicate the size and direction of the ground reaction forces

Participants performed gait analysis 6, 12, and 24 months after injury (Fig. 1). Gait analysis was performed with a marker-based camera system, with 16 high-speed video cameras (Qualisys 7 + , Qualisys AB, Gothenburg, Sweden). Four force plates (AMTI Optima OPT, Watertown, MA, USA) were used to measure ground reaction forces simultaneously with motion data. Twenty reflective markers were placed on anatomical landmarks on the trunk, pelvis, and lower extremity according to a pre-defined marker set [17]. Reflective markers were located bilaterally on the following anatomical landmarks: bilateral acromia; superior anterior iliac spine; superior to patella; knee joint line; tuberositas tibiae; lateral malleoli; second metatarsal head and calcaneus. Single markers were placed on the sternum, sacrum and spinal processes at Th 2 and Th 12, respectively.

Data collection started with a static measurement in the calibrated volume followed by the dynamic movement measurements. Participants were instructed to walk at a self-selected speed along a 10 m distance, until six approved trials were performed. A trial was not approved if the participant didn't place their entire foot on the force plate, if both left and right feet were on the same force plate, or if only one foot touched some of the force plates during the attempt. Participants walked barefoot in underwear and/or shorts. No walking aids were used during the data collection. All approved gait trials were included in the data processing.

### Data processing

Joint kinematics and kinetics were calculated in Visual 3D (version 2023.04.3, C-Motion, Inc., Germantown, USA) before exporting to Excel (2016). In Visual 3D, the prevalence of continuity of all data was confirmed with respect to; marker placement, force plate strikes, and ensuring each gait trial represented a complete gait cycle. During this process, an additional quality control was conducted, and any identified unapproved walking trials were excluded from further analysis. Consequently, the number of approved walking trials for the study participants had a median of 6 (range 4–6), and for the reference group, a median of 6 (range 3–6).

Data were subsequently extracted as discrete variables for; minimum knee flexion, knee range of motion, extending knee moment, and generated power for the knee. Additionally, normalized values for angles and moments in the sagittal plane for a full gait cycle at the hip, knee, and ankle joints were obtained, alongside non-normalized values for absorbed and generated power in the knee joint.

The cumulative sum of knee power was calculated by first obtaining each individual value from each frame for all approved gait trials. Then, all positive values were summarised to represent the amount of generated power, and all negative values summarised to represent the amount of absorbed power.

The selection of gait variables focused on the sagittal plane, as a previous validation study of the chosen marker set demonstrated that data from motion analysis is most accurate in this plane [18]. Minimal knee flexion was selected due to the well-known difficulty in achieving adequate knee extension following a tibial plateau fracture. Knee range of motion provides an overview of the joint's mobility during gait. Maximal knee extending moment was expected to indicate the physical ability to extend the knee joint in combination with the ability to bear weight through the knee. The cumulative sums of generated and absorbed power around the knee joints aimed to measure and compare the amount of muscle work performed during walking. This is because the quantity of generated and absorbed joint power can be interpreted as representing concentric and eccentric muscle work around the knee joint [19].

### Statistical analysis

The statistical analyses were performed using IBM SPSS Statistics version 29.0 (IBM, Armonk, NY, USA). The significance level was set at *p* < 0.05.

#### Linear mixed-effects model

The linear mixed-effects model was performed to investigate knee joint kinematics and kinetics over time for the six gait variables. The model was primarily utilized to predict the interaction effect between time and knee injury status, which indicated whether the injured and non-injured legs exhibited different developmental patterns over time. In the absence of an interaction effect, the model was subsequently employed to predict the main *effect of knee injury status*, identifying any differences between the injured and the non-injured legs. Additionally, the model assessed the main *effect of time* to determine whether there were any changes between the six-month and the 24-month follow-up.

The model accounted for *the effect of time* and *the effect of knee injury status,* and the interaction between time and knee injury status as a fixed effect. The effect of time was considered as categorical and classified into three categories: 6, 12 and 24 months. This categorization allowed for the potential non-linear effects of time and accommodated the study design, which involved data collection at these specific time points. The effect of knee injury status was also considered as categorical and included two categories – a non-injured leg and an injured leg. Patient-ID was considered a random effect. Expected mean values and 95% confidence intervals (CI) were reported for each model.

To address this, a linear mixed-effect model was employed across six gait variables related to knee joint kinematics and kinetics. The model was primarily supposed to evaluate whether the injured and the non-injured leg developed differently over time, called an interaction effect. In case of there was no different development, legs were assumed to have the same difference across all three time-points. The model was therefore supposed to identify the size of this difference, which was called *the effect of knee injury status*. Additionally, if legs did not exhibit a different development over time and were assumed to have the same difference across all time-points, we wanted to know whether both legs improved or got worsened over time. This was called *the effect of time* and was supposed to identify whether there was a positive or negative change over time.

#### Mann Whitney U-test

The Mann–Whitney U test was performed to identify differences between the non-injured leg and the reference group. This non-parametric test was chosen due to the relatively small sample size of the study participants and because not all variables were shown to be normally distributed according to the Shapiro–Wilk’s test.

## Results

**Fig. 2:**
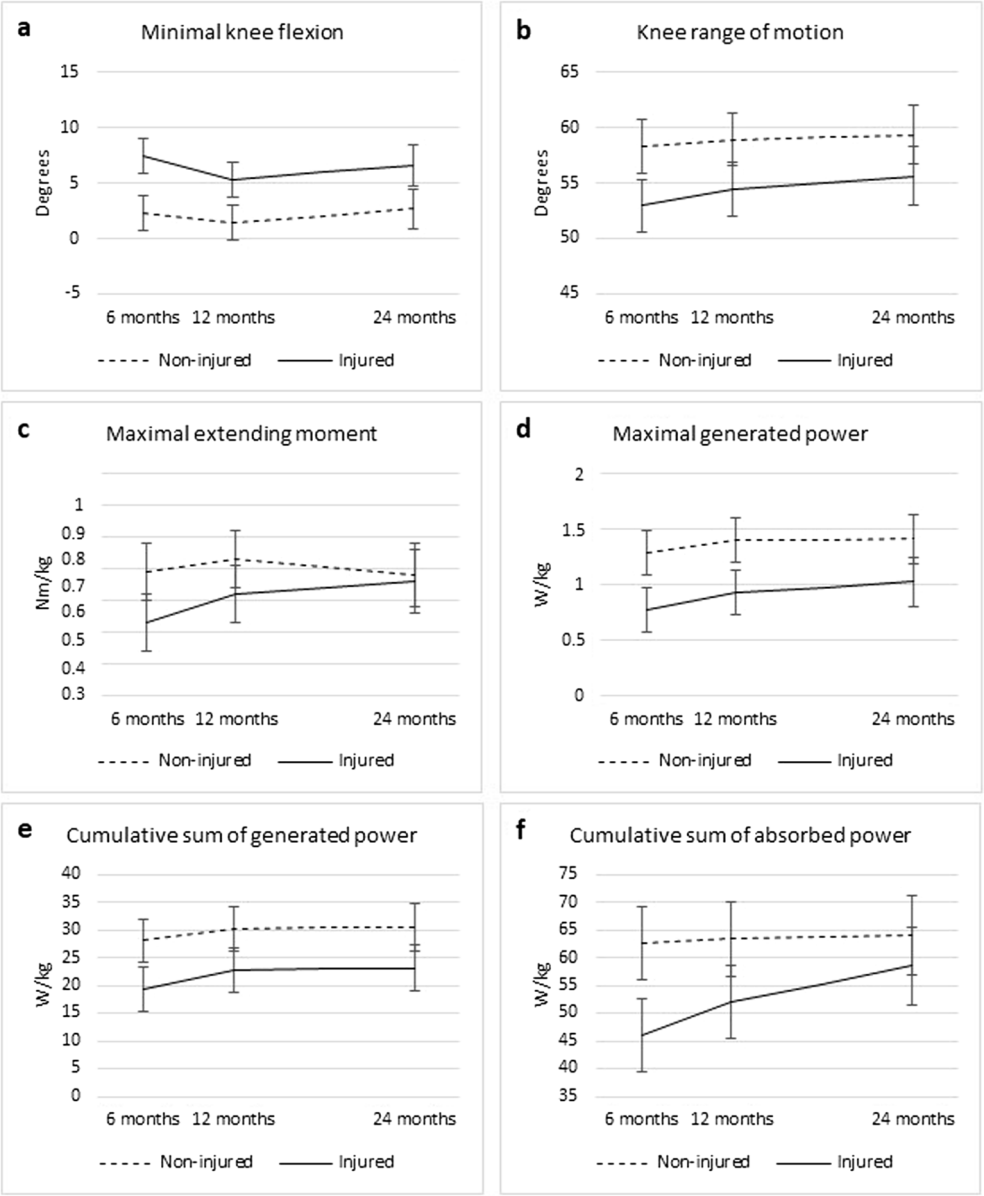
**a-f** Predicted mean values (with 95% confidence intervals) in the knee joint derived from the linear mixed effects model, provided for both the injured and non injured legs at each respective time point

The results obtained from the linear mixed-effects model indicate that the only variable exhibiting a statistically significant interaction effect between time and knee injury status was the cumulative sum of absorbed power (*p* = 0.02), (Table 2, Fig. 2a-f). For this variable, the injured leg exhibited a gradual increase in cumulative absorbed power between the six-month and the 24-month follow-up (Table 3, Fig. 2f), eventually achieving a level comparable to that of the non-injured leg at the 24-month follow-up with no significant differences between the injured and non-injured legs (*p* = 0.06, difference 5.55, 95% CI -0.21 to 11.3).

**Table 2:** *P*-values obtained from the ANOVA table of the linear mixed-effects model: Interaction between the effect of time and the effect of knee injury status and the separate effects of time and knee injury status

	**Linear mixed-effects model**	
**Minimal knee flexion (single stance), degrees**	Interaction effect	**0.61**
	Time	**0.06**
	Knee injury status	**<0.001**
**Knee range of motion (gait cycle), degrees**	Interaction effect	**0.61**
	Time	**0.09**
	Knee injury status	**<0.001**
**Maximal extending moment (single stance), Nm/kg**	Interaction effect	**0.06**
	Time	**0.06**
	Knee injury status	**<0.001**
**Maximal generated power (gait cycle), W/kg**	Interaction effect	**0.68**
	Time	**0.02**
	Knee injury status	**<0.001**
**Cumulative sum of generated power (gait cycle), W/kg**	Interaction effect	**0.78**
	Time	**0.02**
	Knee injury status	**<0.001**
**Cumulative sum of absorbed power (gait cycle), W/kg**	Interaction effect	**0.02**
	Time	**0.03**
	Knee injury status	**<0.001**

**Table 3:** Predicted mean values (with 95% confidence intervals) for the knee joint derived from the linear mixed-effects model, provided for the injured and non-injured legs at each respective time-point

Time point	**Non-injured** Mean value (95% CI)	**Injured** Mean value (95% CI)
**Minimal knee flexion (single stance), degrees**		
6	2 (1—4)	7 (6—9)
12	1 (0—3)	5 (4—7)
24	3 (1—5)	7 (5—9)
**Knee range of motion (gait cycle), degrees**		
6	58 (56—61)	53 (51—55)
12	59 (57—61)	54 (52—57)
24	59 (57—62)	56 (53—58)
**Maximal extending moment (single stance), Nm/kg**		
6	0.69 (0.60—0.78)	0.53 (0.44—0.62)
12	0.73 (0.63—0.82)	0.62 (0.53—0.71)
24	0.68 (0.57—0.78)	0.66 (0.56—0.76)
**Maximal generated power (gait cycle), W/kg**		
6	1.28 (1.08—1.48)	0.77 (0.57—0.97)
12	1.40 (1.20—1.60)	0.93 (0.73—1.13)
24	1.41 (1.19—1.63)	1.02 (0.80—1.24)
**Cumulative sum of generated power (gait cycle), W/kg**		
6	28.06 (24.13—31.99)	19.32 (15.39—23.25)
12	30.13 (26.17—34.09)	22.80 (18.83—26.76)
24	30.58 (26.34—34.82)	23.19 (18.96—27.43)
**Cumulative sum of absorbed power (gait cycle), W/kg**		
6	62.57 (55.94—69.20)	46.06 (39.43—52.69)
12	63.36 (56.69—70.03)	52.00 (45.33—58.68)
24	64.11 (57.05—71.18)	58.57 (51.51—65.63)

The remaining five variables did not exhibit a significant interaction effect (Table 2, Fig. 2a-e). Consequently, both legs demonstrated similar changes between the six-month and the 24-month follow-up for the following variables: minimal flexion in the knee joint during single stance, knee joint range of motion, extending knee moment, maximal generated power, and cumulative sum of generated power (Table 3, Fig. 2a-e).

For all variables that did not show an interaction effect, each variable demonstrated a significant effect of knee injury status (*p* < 0.001), indicating a distinction between the injured and the non-injured legs (Tables 2 and 3). The injured leg demonstrated a difference of 4° (95% CI 3–5) in terms of deficit in knee joint extension during single stance compared to the non-injured leg. Additionally, the range of motion in extension-flexion was 5° (95% CI 3–6) less in the injured leg than in the non-injured leg.

Two variables demonstrated a separate significant effect of time: the maximal generated power (mean difference 0.19, 95% CI 0.05 – 0.33) and the cumulative sum of generated power (mean difference 3.19, 95% CI 0.62–5.71) (Tables 2 and 3).

Taken together, the injured leg demonstrated improvements between the six-month and 24-month follow-up periods in terms of the cumulative sum of absorbed power compared with the non-injured leg. A significant difference was observed between the injured and non-injured legs across all variables. A progressive improvement between the six-month and 24-month follow-up periods was observed for the maximal generated power and the cumulative sum of generated power. However, minimal knee flexion, knee range of motion and maximal extending knee joint moment did not show any significant changes between the six-month and 24-month follow-up periods.

Moreover, based on the observed means of the kinetic variables (Table 4), a percentage limb asymmetry was computed at the 24-month follow-up. These percentages indicated that the maximum extending knee joint moment and the cumulative sum of absorbed power reached 90% in limb asymmetry. In contrast, the maximum generated power was only 58%, and the cumulative sum of generated power was 67%.

**Table 4:** Observed kinematics and kinetics from the gait analysis for the injured and the non-injured knee joint for the study group and for controls. Data are shown as median (interquartile range)

Time point	**Non-Injured** Median (IQR)	**Injured** Median (IQR)	**Controls** Median (IQR)	*p*-value^a^
**Minimal knee flexion (single stance), degrees**				
6	2 (8)	8 (6)		
12	0 (6)	5 (5)		
24	2 (4)	6 (7)	0 (3)	0.061
**Knee range of motion (gait cycle), degrees**				
6	58 (9)	53 (9)		
12	58 (10)	55 (9)		
24	59 (6)	56 (9)	59 (7)	0.686
**Maximal extending moment (single stance), Nm/kg**				
6	0.67 (0.25)	0.53 (0.30)		
12	0.70 (0.25)	0.60 (0.40)		
24	0.74 (0.24)	0.67 (0.33)	0.49 (0.36)	**0.003**
**Maximal generated power (gait cycle), W/kg**				
6	1.21 (0.92)	0.62 (0.54)		
12	1.35 (0.77)	0.79 (0.77)		
24	1.43 (0.62)	0.83 (0.71)	1.29 (1.01)	0.700
**Cumulative sum of generated power (gait cycle), W/kg**				
6	25.64 (12.58)	18.24 (14.02)		
12	29.46 (14.96)	19.70 (19.26)		
24	32.13 (14.90)	21.47 (12.74)	25.49 (14.01)	0.233
**Cumulative sum of absorbed power (gait cycle), W/kg**				
6	61.01 (10.37)	45.26 (20.83)		
12	62.09 (22.16)	49.42 (17.82)		
24	64.26 (20.68)	59.49 (17.02)	57.89 (17.56)	0.263

^a^Comparison between the non-injured side from the 24-months follow-up and controls using Mann Whitney U-test

According to the Mann Whitney U-test conducted between the non-injured leg and the control group during the 24-months follow-up, only one variable exhibited a significant difference. The maximal extending moment in the knee joint was higher in the non-injured leg compared with the control group (*p* = 0.03). None of the other variables showed any significant differences between the non-injured leg and the control group (Table 4).

**Fig. 3:**
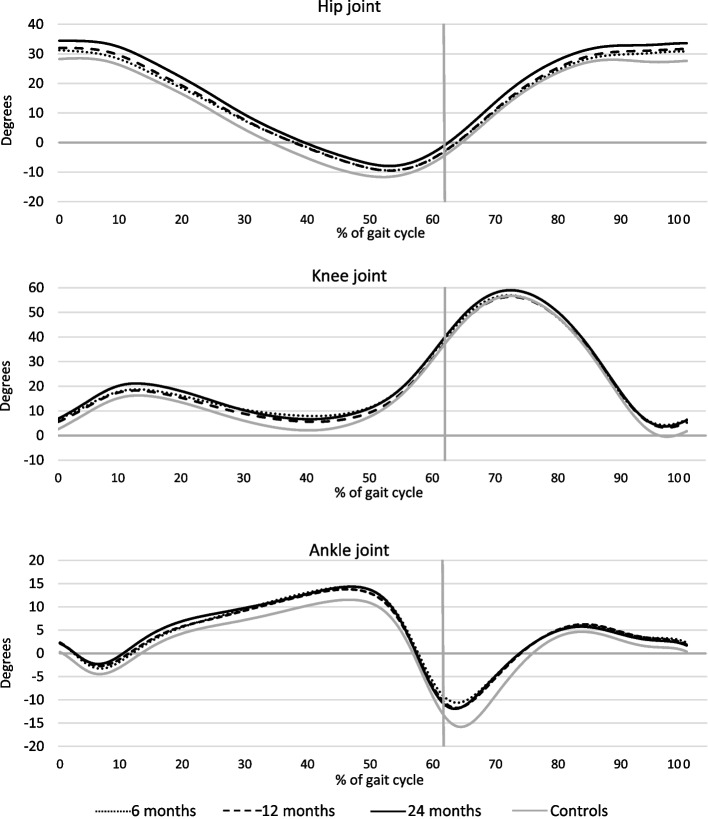
Mean of joint angles (degrees) for the injured leg and for the control group in the sagittal plane throughout the gait cycle. Hip joint (-extension/ + flexion). Knee joint (-extension/ + flexion). Ankle joint (-plantarflexion/ + dorsiflexion). The vertical grey lines define the transition from stance phase to swing phase

**Fig. 4:**
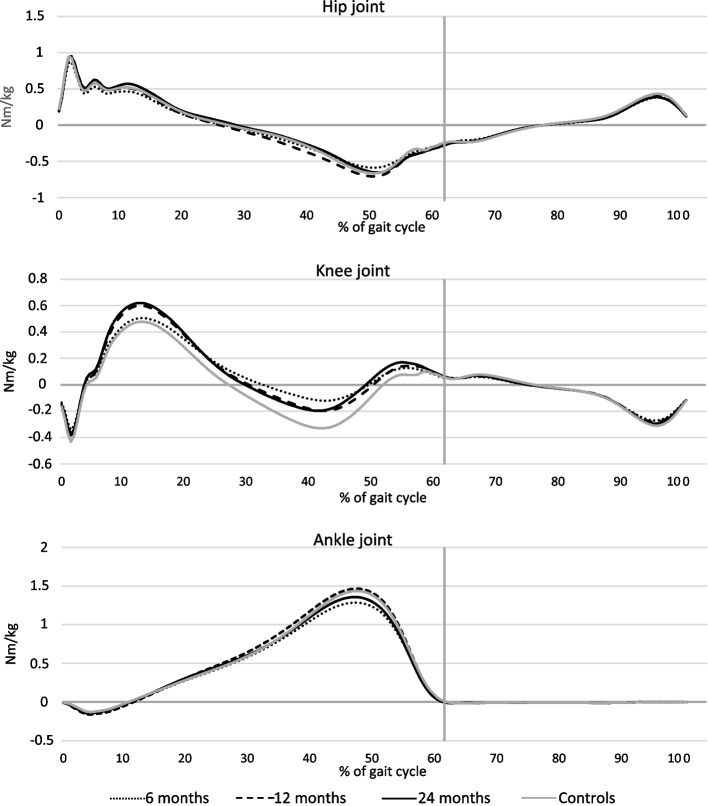
Mean of joint moments (Nm/kg) for the injured leg and for the control group in the sagittal plane throughout the gait cycle. Hip joint (-flexing/ + extending). Knee joint (-flexing/ + extending). Ankle joint (-dorsal flexing/ + plantar flexing). The vertical grey lines define the transition from stance phase to swing phase

**Fig. 5:**
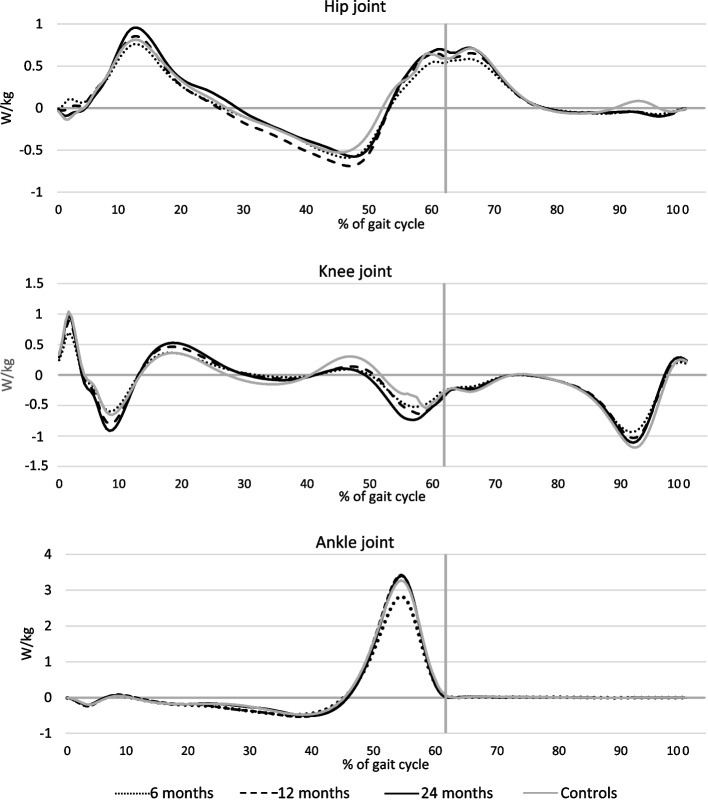
Mean of generated (positive) and absorbed (negative) power (W/kg) for the injured leg and for the control group in the sagittal plane throughout the gait cycle. The vertical grey lines define the transition from stance phase to swing phase

The mean values of joint angles, joint moments, and joint power in the sagittal plane for the hip joint, knee joint and ankle joint, normalized to a gait cycle (0–100%), are visualized for the injured leg at each time-point, and for the controls corresponding to the 24-months study group in Figs. 3, 4 and 5. The normalization to a gait cycle allows for comparisons between groups.

## Discussion

The most clinically important findings of this study revealed that even two years following a tibial plateau fracture, patients continued to demonstrate worse ability to extend the knee joint during single stance along with lower generation of power during walking. Improvements in the production of power within the injured leg were observed over time, while no significant advancements were noted in knee kinematics and maximal extending knee joint moment. Moreover, at the 24-months follow-up, the non-injured leg performed similarly compared with a healthy control-group.

The primary objective of this study was to investigate knee joint kinematics and kinetics during gait following a tibial plateau fracture using a linear mixed-effects model. The second aim of the study was to compare the non-injured leg to a healthy control group to determine whether the non-injured leg exhibited any effects of the injury two years post-injury.

According to the linear mixed-effects model, the only variable showing an interaction effect was the cumulative sum of absorbed power. This suggests that the injured leg improved over time and eventually produced an equivalent amount of eccentric muscle work as the non-injured leg by the 24-month follow-up. For the remaining variables, the differences observed between the legs at the six months follow-up persisted at the 24-months follow-up.

Minimal knee flexion, range of motion and maximal knee extending moment demonstrated a significant side difference with poorer outcomes for the injured leg compared to the non-injured leg. None of these variables changed in-between the legs or over time from the six months follow-up to the 24-months follow-up.

Bennett et al. who investigated longitudinal kinematic changes over time following surgically treated tibial plateau fractures, observed improvements in peak knee flexion during mid-swing from two weeks to two years after surgery [13]. Additionally, Bennett et al. noted the greatest improvement during the first six months, which aligns with the present study's findings, suggesting that from six months onwards, there are no significant changes in knee joint kinematics during walking.

The kinematic variables from the present study exhibited side differences of approximately five degrees, which could thus be considered within the margin of measurement error. Despite this, we believe it is valuable to highlight the side difference in minimal knee flexion during single stance, as problems with passive and active knee extension have been observed in clinic in patients with tibial plateau fractures. Additionally, marker-based motion analysis systems have been shown to underestimate knee flexion depending on the task performed [18, 20, 21]. With that in mind, we emphasize the study's results, which indicate that some participants were unable to fully extend the injured knee joint when bearing full body weight on the same leg, for reasons that warrant further investigation.

Regarding the maximal extending knee joint moment, we did not observe any improvement from six months onwards. These results are consistent with the findings of Millar et al., who studied peak joint moments in the hip, knee, and ankle joints from two weeks after the index injury to two years after surgically treated tibial plateau fractures [14]. They did however, find improvements in peak knee joint moments between three to six months post-injury, with minimal progress thereafter. Additionally, at two years, the study group showed lower values compared with healthy controls [14].

This study is the first to present variables regarding knee joint power over time, which makes comparisons with previous studies challenging. Interestingly, both maximal generated power and the cumulative sum of generated power showed improvements over time. However, there were no interaction effects for the generated power, and the side difference between legs persisted at the 24-month follow-up. Additionally, a greater production of absorbed power than generated power was observed. The latter is normal [19]; however, the proportion of concentric power was significantly lower in the injured leg compared with the non-injured leg with a leg asymmetry of 58% in maximal generated power and 67% in the cumulative sum of generated power. We interpret these findings to indicate that both eccentric and concentric muscle work increase over time from six months to 24 months after injury. Nevertheless, a significantly lower production of concentric muscle work persisted two years after the injury.

Previous literature has demonstrated that a significant proportion of patients with tibial plateau fractures struggle to return to more demanding physical activities [22–25]. Although this study did not aim to investigate the return to previous levels of sports or exercise, the difficulty in resuming activities such as running or jumping was frequently mentioned during the gait analysis visits. The study's findings on the reduced ability to generate power, with approximately 60% in limb asymmetry, do not provide an explanation or solution for the participants' inability to run or jump, but they may offer some insight into the patient's challenges. A common clinical threshold for limb asymmetry before returning to more demanding sports activities is that the injured leg should achieve at least 90% of the capacity of the healthy leg, and in this comparison, 60% is very low. Moreover, considering that these disparities occur during walking, we can imagine that the asymmetry would be even greater during activities involving higher speeds. In the future, it would be interesting to conduct a study that investigates why participants have difficulty generating power and develops solutions to improve this issue.

In summary, from the findings from the linear mixed-effects model, we infer that restoring the ability to extend and flex the knee joint after six months post-injury is challenging, while improving the degree of power development over time appears to be more feasible. Side differences, indicating superior results for the non-injured leg, were observed at all time points for all variables, except for absorbed power, which showed no side difference at the 24-month follow-up.

The second objective of the present study aimed to assess the impact of the injury on the non-injured leg by comparing it with a healthy control group during the 24-months follow-up. It is commonly believed that variations after an injury can have a negative impact on the contralateral limb. However, findings from this study suggest that the non-injured knee joint is not significantly affected by the injury. The results revealed a significant difference only in the extending knee joint moment, with a higher mean moment in the non-injured leg compared with the control group. It is possible that this increased extending knee joint moment could contribute to a greater loading, potentially leading to an increased risk of injuries on the contralateral side. Nonetheless, additional research is warranted to answer the mechanism behind the higher amount of knee extending moment in the non-injured leg. Additional research is also warranted to draw conclusions regarding potential affection in other joints due to changes in loading and movement patterns after an injury.

Based on both current and prior research findings, we emphasize the importance of focusing on the patient’s movement function in the initial phases following injury. Subsequently, a comprehensive approach should be employed, involving training of both knee flexors and extensors to improve strength, proprioception, reaction, and ideally enhance power generation during gait. We share this viewpoint with Millar et al., who also highlighted the critical period of three to six months for knee joint rehabilitation [14], as well as with Iliopoulos et al., who suggested focus on quadriceps strength training and proprioception during rehabilitation [6]. In the context of gait, the main contributors to driving the body forward are the muscles of the plantar flexors and hip extensors [19, 26]. Nevertheless, the quadriceps muscles play an important role, particularly in regulating limb stiffness during the stance phase [26].

This study had some limitations. First, a larger sample size would have been advantageous for drawing more robust conclusions and conducting subgroup analyses. An increased sample could also have allowed for the development of more detailed statistical models, potentially including joints other than the knee. We hypothesize that compensation patterns for the injured knee might occur in both the ipsilateral and contralateral hip and ankle joints. This hypothesis is supported by Bennett et al., who observed differences in joint kinematics from healthy references at two years, especially at the ankle joint [13]. Additionally, another limitation is the lack of information about the rehabilitation protocols followed by the participants post-injury. Standardizing rehabilitation across participants may be difficult due to the necessity of tailoring treatment to individual needs.

## Conclusion

Two years after a tibial plateau fracture, participants exhibited difficulties in knee extension during single stance, as well as low generation of power around the injured knee joint during walking. Although power production improved over time, there were no improvements in kinematic variables from six months after injury onwards. In terms of knee joint kinematics and kinetics, the non-injured leg performed comparably to healthy references two years after injury.

## Data Availability

The datasets used and/or analysed during the current study are available from the corresponding author on reasonable request.

## Abbreviations

ROM: Range of motion
AO/OTA: AO foundation/Orthopaedic Trauma Association
SFR: Swedish Fracture Register
IQR: Inter quartile range
BMI: Body mass index
CI: Confidence Interval

## References

[1] Rohra N, Suri HS, Gangrade K. Functional and Radiological Outcome of Schatzker type V and VI Tibial Plateau Fracture Treatment with Dual Plates with Minimum 3 years follow-up: A Prospective Study. J Clin Diagn Res. 2016;10(5):RC05-10. 27437315 10.7860/JCDR/2016/18732.7855 PMC4948491

[2] Giannotti S, Giovannelli D, Dell’Osso G, Bottai V, Bugelli G, Celli F, et al. Angular stable plates in proximal meta-epiphyseal tibial fractures: study of joint restoration and clinical and functional evaluation. Musculoskelet Surg. 2016Apr;100(1):15–8. 26590578 10.1007/s12306-015-0389-5

[3] Jansen H, Frey SP, Doht S, Fehske K, Meffert RH. Medium-term results after complex intra-articular fractures of the tibial plateau. Journal of orthopaedic science : official journal of the Japanese Orthopaedic Association. 2013Jul;18(4):569–77. 23661179 10.1007/s00776-013-0404-3

[4] Elsoe R, Larsen P, Shekhrajka N, Ferreira L, Ostgaard SE, Rasmussen S. The outcome after lateral tibial plateau fracture treated with percutaneus screw fixation show a tendency towards worse functional outcome compared with a reference population. European journal of trauma and emergency surgery : official publication of the European Trauma Society. 2016Apr;42(2):177–84. 26038018 10.1007/s00068-015-0497-9

[5] Ebrahimzadeh MH, Birjandinejad A, Moradi A, Fathi Choghadeh M, Rezazadeh J, Omidi-Kashani F. Clinical instability of the knee and functional differences following tibial plateau fractures versus distal femoral fractures. Trauma monthly. 2015Feb;20(1): e21635. 25825697 10.5812/traumamon.21635 PMC4362032

[6] Iliopoulos E, Agarwal S, Khaleel A. Walking impairments after severe tibia plateau fractures. A gait pattern analysis. J Orthop Sci. 2020;25(2):276–8. 30962098 10.1016/j.jos.2019.03.015

[7] Elsoe R, Larsen P. Asymmetry in gait pattern following bicondylar tibial plateau fractures-A prospective one-year cohort study. Injury. 2017Jul;48(7):1657–61. 28479051 10.1016/j.injury.2017.04.045

[8] Warschawski Y, Elbaz A, Segal G, Norman D, Haim A, Jacov E, et al. Gait characteristics and quality of life perception of patients following tibial plateau fracture. Arch Orthop Trauma Surg. 2015Nov;135(11):1541–6. 26386838 10.1007/s00402-015-2325-4

[9] Warschawski Y, Drexler M, Batko B, Elias S, Goldstein Y, Frenkel Rutenberg T, et al. Correlation between preoperative imaging parameters and postoperative basic kinematics-based functional outcome in patients with tibial plateau fractures. Clin Biomech (Bristol, Avon). 2019May;65:87–91. 31005694 10.1016/j.clinbiomech.2019.04.009

[10] Elsoe R, Larsen P. Delayed but favourable outcome of lateral tibial plateau fracture after screw fixation: A 3-year prospective cohort study of 56 patients. Knee. 2021Mar;29:280–90. 33677152 10.1016/j.knee.2021.02.015

[11] Larsen P, Traerup J, Mikuzis M, Elsoe R. Patient-reported and Functional Outcomes of Bicondylar Tibial Plateau Fractures Managed by Internal Screw Fixation in Combination with An Ilizarov Fixator: A Case Series of 22 Patients with Long-term Follow-up. Strategies Trauma Limb Reconstr. 2019;14(2):85–91. 32742419 10.5005/jp-journals-10080-1432 PMC7376587

[12] Deleanu B, Prejbeanu R, Crisan D, Predescu V, Popa I, Poenaru DV. Gait characteristics before hardware removal in patients operated upon for tibial plateau fractures. Int Orthop. 2015Jul;39(7):1411–5. 25673510 10.1007/s00264-015-2691-0

[13] Bennett KJ, Millar SC, Fraysse F, Arnold JB, Atkins GJ, Solomon LB, et al. Postoperative lower limb joint kinematics following tibial plateau fracture: A 2-year longitudinal study. Gait Posture. 2021Jan;83:20–5. 33069125 10.1016/j.gaitpost.2020.10.005

[14] Millar SC, Bennett K, Fraysse F, Arnold JB, Solomon LB, Thewlis D. Longitudinal changes in lower limb joint loading up to two years following tibial plateau fracture. Gait Posture. 2020May;78:72–9. 32272398 10.1016/j.gaitpost.2020.03.008

[15] Fändriks A, Tranberg R, Karlsson J, Möller M, Zügner R. Gait biomechanics in patients with intra-articular tibial plateau fractures - gait analysis at three months compared with age- and gender-matched healthy subjects. BMC Musculoskelet Disord. 2021Aug 17;22(1):702. 34404375 10.1186/s12891-021-04577-y PMC8369713

[16] Marsh JL, Slongo TF, Agel J, Broderick JS, Creevey W, DeCoster TA, et al. Fracture and dislocation classification compendium - 2007: Orthopaedic Trauma Association classification, database and outcomes committee. J Orthop Trauma. 2007;21(10):S1-133. 18277234 10.1097/00005131-200711101-00001

[17] Weidow J, Tranberg R, Saari T, Karrholm J. Hip and knee joint rotations differ between patients with medial and lateral knee osteoarthritis: gait analysis of 30 patients and 15 controls. Journal of orthopaedic research : official publication of the Orthopaedic Research Society. 2006Sep;24(9):1890–9. 16838360 10.1002/jor.20194

[18] Tranberg R, Saari T, Zügner R, Kärrholm J. Simultaneous measurements of knee motion using an optical tracking system and radiostereometric analysis (RSA). Acta Orthop. 2011Apr;82(2):171–6. 21463221 10.3109/17453674.2011.570675 PMC3235287

[19] Winter DA. Energy generation and absorption at the ankle and knee during fast, natural, and slow cadences. Clin Orthop Relat Res. 1983May;175:147–54. 6839580

[20] Wang W, Li X, Zhang T, Li J, Viellehner J, Komnik I, et al. Effects of soft tissue artifacts on the calculated kinematics of the knee during walking and running. J Biomech. 2023Mar;150: 111474. 36871431 10.1016/j.jbiomech.2023.111474

[21] Tsai TY, Lu TW, Kuo MY, Lin CC. Effects of soft tissue artifacts on the calculated kinematics and kinetics of the knee during stair-ascent. J Biomech. 2011Apr 7;44(6):1182–8. 21296352 10.1016/j.jbiomech.2011.01.009

[22] O’Neill DC, Sato EH, Myhre LA, Kantor AH, Rothberg DL, Higgins TF, et al. Return to Skiing After Tibial Plateau Fracture. Orthop J Sports Med. 2023Oct;11(10):23259671231205924. 37868212 10.1177/23259671231205925 PMC10585993

[23] Robertson GAJ, Wong SJ, Wood AM. Return to sport following tibial plateau fractures: A systematic review. World journal of orthopedics. 2017Jul 18;8(7):574–87. 28808629 10.5312/wjo.v8.i7.574 PMC5534407

[24] Kraus TM, Martetschlager F, Muller D, Braun KF, Ahrens P, Siebenlist S, et al. Return to sports activity after tibial plateau fractures: 89 cases with minimum 24-month follow-up. Am J Sports Med. 2012Dec;40(12):2845–52. 23118120 10.1177/0363546512462564

[25] Quintens L, Van den Berg J, Reul M, Van Lieshout E, Nijs S, Verhofstad M, et al. Poor sporting abilities after tibial plateau fractures involving the posterior column: how can we do better? European journal of trauma and emergency surgery : official publication of the European Trauma Society. 2021Feb;47(1):201–9. 31473772 10.1007/s00068-019-01220-3

[26] Farris DJ, Sawicki GS. The mechanics and energetics of human walking and running: a joint level perspective. J R Soc Interface. 2012Jan 7;9(66):110–8. 21613286 10.1098/rsif.2011.0182 PMC3223624

